# Naming no names: Comments on the taxonomy of small piroplasmids in canids

**DOI:** 10.1186/s13071-016-1567-5

**Published:** 2016-05-18

**Authors:** D. James Harris

**Affiliations:** Centro de Investigação em Biodiversidade e Recursos Genéticos (CIBIO), Universidade do Porto, InBIO Laboratório Associado. Campus Agrário de Vairão, Vairão, Porto, 4485-661 Portugal; Departamento de Biologia, Faculdade de Ciências, Universidade do Porto, R. Campo Alegre s/n, Porto, 4169-007 Portugal

**Keywords:** *Babesia*, *Theileria*, Phylogeny, *Vulpes*

## Abstract

Based on phylogenetic analyses, various taxonomic changes have recently been proposed for tick-transmitted small piroplasmids, including descriptions of new species. It is however essential that any such changes comply with the International Code of Zoological Nomenclature. Unfortunately, this has not been the case, and some recently proposed names are therefore invalid. The use of informal clade names is necessary until formal valid descriptions are available.

## Letter to the editor

Taxonomists have two primary roles, to name organisms and to classify them. This hierarchical system dates back to Linnaeus, and in zoology is governed by the International Code of Zoological Nomenclature [1] (hereafter the *Code*). While the *Code* has strict rules, flexibility is essential. Taxonomy is expected to reflect phylogeny, and recent advances in this field, especially the employment of molecular tools, have led to many reclassifications and reassignments of species, e.g. [2]. However, all such taxonomic changes must follow the *Code* to be valid scientific names.

Recently Baneth et al. [3] carried out a phylogenetic analysis that clearly showed that a tick-transmitted small piroplasmid that infects dogs and foxes and named *Theileria annae* by Zahler et al. [4], is not actually related to other *Theileria* Bettencourt, Franca & Borges, 1907, but rather is allied with *Babesia* spp. from carnivores (sometimes referred to as “*B. microti*-related”), in turn sister taxa to *Babesia microti* (Franca, 1910). Other molecular assessments have led to similar conclusions, e.g. [5]. Therefore, Baneth et al. [3] proposed the renaming of *T. annae* as *Babesia vulpes* Baneth, Florin-Christensen, Cardoso & Schnittger, 2015. However, under the principle of priority, the valid name is the oldest available. The recognition of *T. annae* as a member of *Babesia* Starcovici, 1893 does not affect the species name, and *Babesia annae* would be the correct scientific name. Furthermore, the *Code* (Article 16.4) clearly states that “every new specific and subspecific name published after 1999 … must be accompanied in the original publication by the explicit fixation of a holotype”. Additionally when the holotype or syntype are extant specimens, they must be deposited in a collection, with a given name and locality (Article 16.4.2) and, since 1999, all names (including replacement names) must be explicitly indicated as intentionally new (Article 16.1). Finally, Article 13.1.1 requires that new names are accompanied by a description or definition that states in words characters that are purported to differentiate the taxon. Given that Baneth et al. [3] conform to only one of these requirements (i.e. the species name was clearly indicated as new and registered in ZooBank, see [3]), *Babesia vulpes* must be considered *nomen nudum* and therefore is an unavailable name.

Unfortunately the taxonomic situation is more complicated, because Zahler et al. [4] in the original reference to *T. annae* also failed to identify either a hapantotype (a hapantotype is a series of related individuals of protists, and the entire series acts as the nomenclatural type of the species, that is the entire series has the function of the holotype) or give an appropriate description. *Theileria annae* must also therefore be considered a *nomen nudum* and an unavailable name.

Various members of *Babesia* have been identified in European foxes (members of the genus *Vulpes* Frisch, 1775), including “*Babesia annae*” [6], a member of the “*B. microti* group”, and *Babesia cani*s Piana & Galli-Vallerio, 1895 [7], while in North Africa another lineage of *Babesia* in foxes was identified, related to *Babesia conradae* Kjemtrup, Wainwright, Miller, Penzhorn & Carreno, 2006 [8]. Clearly, specific diagnoses and defining a type are essential when dealing with multiple unrelated parasites infecting the same hosts, otherwise it is not clear which name is appropriate for a given parasite.

Based on the sequence of the whole genome of *B. microti*, it was suggested that this species is “significantly distant from all species of Babesidae [*sic*] and Theileridae [*sic*], and defines a new clade” [9]. It seems likely therefore, that both the generic and specific names of other taxa within this group will alter in the future. The “microti group” also includes parasites from badgers *Meles meles* (Linnaeus, 1758), and “*T. annae*” has recently been isolated from the tick *Ixodes canisuga* Johnston, 1849 from badgers [10]. The complete host range of this group is therefore not yet clear. Given that no current valid name is available, such parasites can only be referred to informally as members of the “microti group”, pending a formal description. Use of such an informal term avoids taxonomic instability through the use of inappropriate scientific names until enough data are gathered to formally describe the species and place them within a stable taxonomic framework.
